# On the Scaling of Transport Phenomena at a Monotonously Changing Hydraulic Conductivity Field

**DOI:** 10.3390/e26110904

**Published:** 2024-10-24

**Authors:** Yaniv Edery, Shaul Sorek

**Affiliations:** 1 Faculty of Civil and Environmental Engineering, Technion, Haifa 32000, Israel; 2 Zuckerberg Institute for Water Research, J. Blaustein Institutes for Desert Research, Ben-Gurion University of the Negev, Midreshet Ben-Gurion 8499000, Israel; sorek@bgu.ac.il

**Keywords:** flow and transport through a monotonously stratified porous medium, Lagrangian particle tracking (LPT), stochasticity and dispersion of transport through a monotonously stratified porous medium

## Abstract

Monotonously stratified porous medium, where the layered medium changes its hydraulic conductivity with depth, is present in various systems like tilled soil and peat formation. In this study, the flow pattern within a monotonously stratified porous medium is explored by deriving a non-dimensional number, $F_{hp}$, from the macroscopic Darcian-based flow equation. The derived $F_{hp}$ theoretically classifies the flow equation to be hyperbolic or parabolic, according to the hydraulic head gradient length scale, and the hydraulic conductivity slope and mean. This flow classification is explored numerically, while its effect on the transport is explored by Lagrangian particle tracking (LPT). The numerical simulations show the transition from hyperbolic to parabolic flow, which manifests in the LPT transition from advective to dispersive transport. This classification is also applied to an interpolation of tilled soil from the literature, showing that, indeed, there is a transition in the transport. These results indicate that in a monotonously stratified porous medium, very low conducting (impervious) formations may still allow unexpected contamination leakage, specifically for the parabolic case. This classification of the $F_{hp}$ to the flow and transport pattern provides additional insight without solving the flow or transport equation only by knowing the hydraulic conductivity distribution.

## 1. Introduction

The transport of dissolved chemicals in saturated porous media and geologically fractured media has been the subject of many studies aimed at characterizing the dispersion of these chemicals.

This dispersion in a deterministic continuum framework can be modeled in an isotropic and homogenous medium by averaging the hydraulic conductivity field (as derived from the intrinsic permeability) over a representative elementary volume (REV) yielding the advection–dispersion equation (ADE) with a Fickian dispersion [1,2]. However, in the past decades, many experimental [3,4,5,6], theoretical [7,8,9,10,11,12,13], and numerical [14,15,16,17,18] studies showed that the omni-present heterogeneity leads to non-Fickian transport, where low concentrations of contaminant are present at long time scales [19,20,21]. While the ADE cannot capture transport in heterogeneous pores or randomly distributed permeability [22,23,24,25], its applicability on stratified heterogeneous porous medium where the permeabilities vary monotonically perpendicular to the pressure gradient [26] was not systematically studied. In what follows, the structural heterogeneity of porous media and its effect on the flow regime leading to the transport mechanism is studied on the basis of rigorous theoretical formalism.

Heterogeneity in the form of monotonically varying hydraulic conductivity at the Darcy scale has received less attention over the years. This form of heterogeneity is non-isotropic by definition [27,28,29,30] and considered as directional hydraulic conductivity if the permeability change aligns with the transport direction [31,32]. Anisotropy in hydraulic conductivity can also be found in soil due to tillage, or in un-tilled soil due to bio-subsoilers, slope variations, or applied head [33,34,35,36]. Furthermore, this directional hydraulic conductivity is found, e.g., in peat formation [37,38], where the layering and compaction of soil over time leads to a directional monotonic decrease in hydraulic conductivity with the depth of the peat formation, forming a unique transport pattern [27,39,40,41]. While parallel flow and transport in stratified heterogeneous media were considered [42,43,44], [45] (p. 222) for various conditions, monotonically varying stratified porous media, as in peat formation, is still an open question, stressing the need for further investigation [33,46].

In this study, a 1D macroscopic flow equation (FE) based on Darcy’s law for saturated medium is applied on a directional hydraulic conductivity field (K[L/T]), where the source of the heterogeneity is a 1D linear gradient of the permeability ($k[L^{2}]$), while the fluid properties remain constant in space and time. This investigation focuses on the directional hydraulic conductivity field, which is aligned with the flow as this theoretical analysis of consistent trends can be insinuated only from 1D distribution and can be verified with natural examples. A non-dimensional number, $F_{hp}$, is derived, relating the length of the hydraulic head gradient, the slope of the hydraulic conductivity change (mimics permeability as it is solely dependent on it for a prescribed fluid), and the average hydraulic conductivity. This non-dimensional number categorizes the transition from the hyperbolic case, which is known to linearly scale the dispersion with the advection to the less explored parabolic FE, and therefore the dominance of the respective advective to the dispersive components in the solute transport. This transition is apparent in a 1D numerical code solving the FE on a range of hydraulic conductivity slopes and averages, and in a Lagrangian particle tracking (LPT) model that follows a Langevin equation, which provides the transport equation (TE). The $F_{hp}$ is shown to scale with the TE for various Péclet (Pe) values and present complementary attributions between the $F_{hp}$ and Pe non-dimensional numbers. This analysis is repeated for conductivity measurement taken from the literature [33], showing that even for a slightly linear slope, the $F_{hp}$ categorization still holds. The $F_{hp}$ scaling with the FE and how it manifests in the ratio between dispersion and advection in the TE is further explored in the Supplementary Materials.

## 2. Data and Methods

### 2.1. Dimensional Analysis of the Macroscopic Flow Equation for Monotonically Varying Hydraulic Conductivity

The dimensional analysis starts with the Darcy relation on an REV scale, with a velocity vector v of the fluid volume ($V_{f}$), namely ${\phi \vartheta }_{f}\;[L/T]\;$associated with the porosity ϕ while neglecting the porous medium pore scale structural change due to pressurized flow (otherwise developed in Section 1 in the Supplementary Materials):

(1) $$-K\cdot \nabla h={\phi \vartheta }_{f},$$

where h=pρg+∇Z·I denotes the piezometric head composed of the pressure head, ρ denotes the compressible fluid density, g is the gravity acceleration, Z is the prescribed altitude, and I denotes the pressure and the identity tensor. Let us note that K in Equation (1) represents the hydraulic conductivity tensor coefficient with units [L/T] (respectively, length over time) and h the piezometric head with [L] units. The effect of the hydraulic conductivity, K, heterogeneity on flow and transport for a given fluid, can be considered as a magnitude of the permeability coefficient $k[L^{2}]$, which is indicative of the degree of resistance to flow due to the formation of geometrical characteristics. Using the transient fluid mass balance (see Equation (S3) in the Supplementary Materials), Equation (1) takes the known form of a parabolic partial differential equation (PDE) for the FE in a Eulerian form as follows:

(2) Ss∂h∂t=∇·K·∇h−G.

where $S_{s}\;[1/L]$ denotes the specific storage, which is the fluid volume released from storage per existing fully saturated storage volume for a given change in the hydraulic head; and G [1/T] denotes the source term rate that is neglected hereinafter in the steady-state case of Equation (2), and in the numerical examples to follow with no effect on the following development. Note that the Lagrangian form of Equation (2), which addresses a particle velocity, will be developed in the Supplementary Materials (see Equations (S11) and (S12) in the Supplementary Materials). To uncouple the role of monotonic spatial heterogeneity at the Darcy scale, the right-hand side (RHS) of Equation (2) is developed as follows:

(3) $$\nabla \cdot \left(K\cdot \nabla h\right)=\nabla \cdot K\cdot \nabla h+\left(K\cdot \nabla \right)\cdot \nabla h\;,$$

Equation (3) is further developed by expressing each dimensional ($\varphi_{d}$) variable by its product form $\varphi_{d}=\varphi_{t}\varphi_{S}$, in which applying the dimensional analysis on a parameter denotes $\varphi_{t}\left(\equiv {\left\lfloor \;\right\rfloor }_{t}\right)$ and $\varphi_{S}\left(\equiv {\left\lfloor \;\right\rfloor }_{s}\right)$, respectively, the typical (dimensional) and non-dimensional (scalar) magnitudes associated with $\varphi_{d}$. Considering $\varphi_{t}$ in a manner that $\varphi_{S}$ becomes of a unit order of magnitude, the non-dimensional scalar magnitudes of Equation (3) allow us to distinguish between the hyperbolic part ∇·K·∇h and the parabolic part $\left(K\cdot \nabla \right)\cdot \nabla h$:

(4) $$[\nabla \cdot K\cdot \nabla h+\left(K\cdot \nabla \right)\cdot \nabla h]\to {\left\lfloor \frac{K}{L_{h}^{2}}\right\rfloor }_{t}{\left\lfloor \left(\frac{L_{h}\nabla K}{K}\nabla h+\nabla^{2}h\right)\right\rfloor }_{s},\;F_{hp}\equiv \frac{L_{h}\nabla K}{K}=\frac{L_{h}\Delta K}{L_{K}K}$$

where, in Equation (4), $L_{h}[L]$ denotes a typical distance associated with the head difference from which head gradient (∇h) is deduced, while the non-dimensional scalar $h\;(\equiv {\left\lfloor h\right\rfloor }_{s})$ is omitted when considering the full non-dimensional form of Equation (3). ∇K is the divergence of hydraulic conductivity, which in 1D becomes the slope ΔK divided by $L_{K}[L]$, a typical distance associated with the change in K ($∆K/L_{K}$). The head difference in this system is applied over the entire system, so $L_{h}$ is the size of the system. Note that the $F_{hp}$ allows us to estimate the magnitude and contribution of each part in Equation (4) for a prescribed formation characterized by the given values of K and ∇·K.

The expression $\frac{L_{h}\Delta K}{L_{K}K}$ is further analyzed by the magnitude of $O\left(∆K\right)\;$and $O\left(K\right)$, by assigning the conductivity magnitude for K to be the order of the average conductivity ($O\left(K\right)\approx \hat{K}$). As such, for any given range of conductivity divergence (which is ∆K for the 1D case), the $F_{hp}$ notation in Equation (4) takes the following form: $F_{hp}=\frac{L_{h}\Delta K}{L_{K}\hat{K}}$. In view of the Equation (4) magnitude analysis, the left-hand side analysis of Equation (2) takes the following form:

(5) SsThth∂h∂ts=K^Lh2thLh∇KK^∇h+∇2hs

for which its non-dimensional form becomes

(6) SsLh2K^Th∂h∂ts=Lh∇KK^∇h+∇2hs

which allows the approximation to be either hyperbolic or parabolic:

SS∂h∂t≅{(7a)∇·K·∇h,     ∀Fhp≫1(7b)K·∇·∇h,     ∀0<Fhp≪1

**Figure 1:**
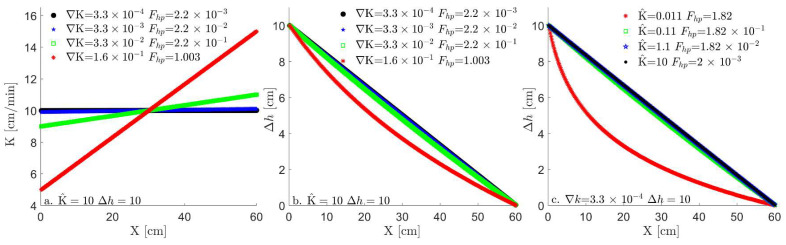
(**a**) Hydraulic conductivity spatial distribution and (**b**) head spatial distribution for a constant hydraulic conductivity value and different slopes. (**c**) Head spatial distributions of different hydraulic conductivity means for a constant slope. Note the transition between the parabolic head (linear function) and hyperbolic (exponential decay of the red curve) as the $F_{hp}$ changes from a fraction of 1 to above 1, respectively.

Note that in Equation (7b), the parabolic form points to the non-linear change in ∆h, in contrast to the linear change in ∆h in the hyperbolic case of Equation (7a), as seen in Figure 1, thus providing a complementary perspective to the analysis for the FE written in Equation (3) [47,48].

For the steady-state case (i.e., subsidence of the transient state), each parameter forming the $F_{hp}$ will differently scale the left-hand side magnitude in Equations (7a) and (7b), and the ratio among the parameters for the case of $F_{hp}\gg 1$ take the following form:

(8) $$\left\|L_{h}\nabla K-\hat{K}\right\|\leq \left\|L_{h}\nabla K\right\|-\left\|\hat{K}\right\|\leq \left\|L_{h}\right\|\left\|\nabla K\right\|-\left\|\hat{K}\right\|\gg 0,$$

which dictates the that $\left\|\nabla K\right\|\gg \left\|\hat{K}\right\|/\left\|L_{h}\right\|$. Equation (8) points to the dominance of the hydraulic conductivity divergence over the hydraulic conductivity mean for the hyperbolic form shown in Equation (7a). This dominance leads to a linear change in ∆h (see Figure 1), and for the 1D case, it can be written as follows:

(9) $$\frac{\nabla K}{\hat{K}}\approx O\left(\frac{∆K/L_{K}}{\hat{K}}\right).\;$$

Recall that $L_{K}$ denotes a typical distance associated with the change in K, and specifically, when K changes linearly it is determined by the domain boundaries. The $L_{K}$ can also be regarded as the correlation length for K change [18], as it establishes the spatial change in the hydraulic conductivity. Thus, in view of Equation (9), assuming that the average conductivity is the same order of magnitude as the conductivity slope [$O\left(∆K\right)=O\left(\hat{K}\right)$], the ratio of LhLK can stand for the $F_{hp}$ value, and thus the case of $F_{hp}\gg 1\;$can be rewritten as follows:

(10a) Lh∇KK^≈OLhLK≫1.

which reflects the interaction between the driving head length scale and the hydraulic conductivity monotonical change as $L_{h}$ governs the extent of ∇h and $L_{K}$ governs the extent of ∇K, respectively. Equation (10a) offers another perspective on the indicators for the transition between hyperbolic to parabolic parts in Equation (4). Similarly, to the order estimation in Equation (10a), for $O\left(L_{K}\right)=O\left(L_{h}\right)$, the possibility for $F_{hp}\gg 1$ can be obtained as follows:

(10b) Lh∇KK^≈O∆KK^≫1,

which reflects the porous medium hydraulic conductivity range in Equation (4). This analysis points to the ratio between the length at which the conductivities and heads vary spatially [as in (10a)], as well as the ratio between the hydraulic conductivity divergence and mean [as in Equation (10b)]. Note that in view of Equations (5) and (9), while $O\left(L_{K}\right)\neq O\left(L_{h}\right)$ and $O\left(∆K\right)\neq O\left(K\right)$, an approximation for $F_{hp}\gg 1$ is obtained as follows:

(10c) $$\frac{L_{h}\nabla K}{\hat{K}}\approx O\left(\frac{L_{h}∆K}{L_{K}\hat{K}}\right)\gg 1⟹\;\frac{∆K}{\hat{K}}\gg \frac{L_{K}}{L_{h}}$$

The non-dimensional analysis of the FE produces a clear distinction between the dominance of the hydraulic conductivity divergence and the hydraulic conductivity mean for the hyperbolic and parabolic case, respectively. However, to understand if and how this distinction manifests in the transport of contaminants, a Lagrangian particle tracking (LPT) model is depicted in the following section.

### 2.2. Lagrangian Particle Tracking Model

To explore how the non-dimensional analysis of the FE manifests in the TE, a well-known LPT code used in previous studies in saturated flow [18,49,50,51] was applied on a 1D hydraulic conductivity slope (which is the divergence in 1D). While both transport and flow can be simulated numerically, transport can also be estimated experimentally in the field. Therefore, within the field scale where contaminant diffusion must be considered for the transport, the parabolic to hyperbolic transition is analyzed for various diffusion magnitudes to the advective, using the Péclet number.

The one-dimensional domain is made of 300 numerical bins with uniform prescribed dimensions and assigned permeabilities that are spatially varying values, from which the local hydraulic conductivity is calculated. Each bin has a size of Δ=0.2 cm, forming the length of the field, L=60 cm. The hydraulic conductivity (K) is varied linearly along the 1D domain to provide a known mean ($\hat{K}$) and slope (∇K) for each fixed head drop (Δh=100,10 or 2 cm) from the inlet to the outlet (see Figure 1 and Table 1 for details). The flow problem for each combination is solved using a finite element model with the Galerkin weighting function [52] to obtain hydraulic head values throughout the domain, which are then converted to velocities (given a porosity ϕ=0.3). At time t = 0, a pulse of 10^6^ particles is introduced (10^7^ and 10^5^ particles showed no significant numerical dispersion) at the inlet and transported between numerical bins using the following Langevin equation:

(11a) $$d=v\left[x\left(t_{k}\right)\right]\delta t+d_{D},$$

where d is the displacement, $x\left(t_{k}\right)$ is the particle’s known location at a time $t_{k}$, v is Darcy’s modulus of fluid velocity v at that location, $\delta t=\frac{\delta s}{v}$ is the temporal displacement magnitude (δs=Δ/10), and $d_{D}$ is the stochastic diffusive displacement equation:

(11b) $$d_{D}=N[0,1]\sqrt{2D_{m}\delta t}.$$

This diffusive displacement is randomly generated from a normal distribution between 0 and 1 (N[0,1]) and multiplied by the square root of the stochastic diffusion coefficient ($D_{m}=10^{-5}\frac{{cm}^{2}}{sec}$) representing the diffusion of ions in water [53]. The local fluid velocity, Equation (1), is obtained as $v_{f}=q\left(x\right)/\phi$, where $q\left(x\right)$ is the local Darcy flux calculated from the local hydraulic conductivity (K(x)) and associated with the local head difference (∆h(x)), which in steady-state flow, Equation (2), reads as follows:

(12) ∇·q(x)=0;   q(x)=−K(x)·∇h(x)

And therefore, from the numerical standpoint, the second derivative for the hydraulic head comes from the change in ∆h between numerical bins due to the change in conductivity.

Following the definition of the diffusion coefficient and Darcy’s modulus of fluid velocity, the Péclet number is directly calculated from its definition as the ratio of advective to diffusive transport rates, given by $Pe=\frac{\delta s}{\sqrt{\frac{4}{\pi }D_{m}\delta t}}$. Using the time duration of a single local jump (δt=δs/v), for a specific particle location with a given velocity v, the Péclet number takes the form of $Pe=\sqrt{\frac{\pi \delta s∆h\hat{K}}{2D_{m}L\phi }}$, as summarized in Table 2 for our set of parameters.

### 2.3. Adapting and Interpolating Conductivity Measurements from Tilled Soil

To test the relevance of the non-dimensional $F_{hp}$ number, a table of hydraulic conductivities measured at various depths and taken at three different periods after soil tillage was taken from the literature [33]. These data were scaled to cm/min so as to align with the LPT model introduced in the previous section (see Table 3 for details). As the table comprised conductivity measurements taken from 3–4 depths for each period, a linear interpolation between depths was performed to produce a continuous range of hydraulic conductivities with depth. The linear interpolation assigned the mean hydraulic conductivity to the first depth value for each depth range, and a linear interpolation was performed between the values. To compare the numerical results, the same total range for each interpolated period was needed, and therefore a pre-determined range of $L_{tot}=60\;cm$ for each period was determined (with the same bin size as in the previous section, Δ=0.2 cm). Then, based on the measurements (recall that in Table 3 not all depth ranges were sampled), the local range was extrapolated according to the following equation:

(13) $$K_{j}={\hat{K}}_{soil}\left(i\right)+\frac{j-\frac{D\left(i\right)}{\Delta }}{\frac{D\left(i+1\right)}{\Delta }}\cdot \frac{{\hat{K}}_{soil}\left(i+1\right)-{\hat{K}}_{soil}\left(i\right)}{(D\left(i+1\right)-D\left(i\right))/L_{tot}},\;\frac{D\left(i\right)}{\Delta }<j\leq \frac{D\left(i+1\right)}{\Delta }$$

where ${\hat{K}}_{soil}\left(i\right)$ is the measured conductivity at depth D(i) and j=1..300 is the index for each interpolated conductivity, $K_{j}$. On the interpolated conductivity field, which coincides with the measured locations, $D\left(i\right)$, the LPT model depicted in the previous section, is applied for Δh=100 and 10.

## 3. Results

### 3.1. Dispersion and the Transition Between the Hyperbolic and Parabolic Flow Equations

**Figure 2:**
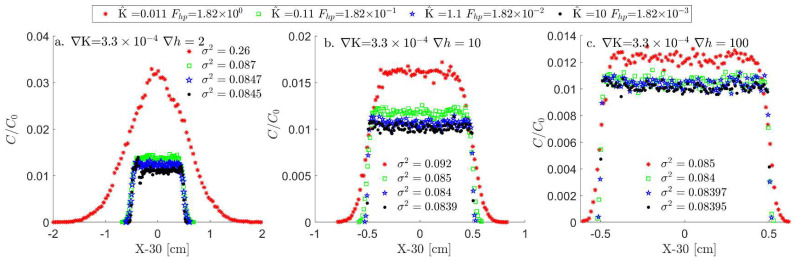
Normalized particle concentration per location, as the first particle j reaches $x_{j}=30$, for a slope of $\nabla K=3.3\times 10^{-4}$; head values of (**a**) 2, (**b**) 10, and (**c**) 100; and varying hydraulic conductivity mean, forming a range of $F_{hp}$’s above and below 1. As can be seen, the variance of the particle distribution ($\sigma^{2}$) has a noticeable change as the $F_{hp}$ is above one. This change is more pronounced for low head values due to the dominance of diffusion time scale on the convective time scale in the Péclet number.

As described in Section 2.1, the non-dimensional number $F_{hp}$ has an associated length scale ($L_{h}),$ which is determined by the system’s boundaries on which the head difference Δh is applied. Through this parameter combination (see Table 1), the transition of the flow equation from hyperbolic to parabolic clearly follows the transition of $F_{hp}>1$ to $F_{hp}<<1$, as depicted in Figure 1a,b. This transition is also manifested in the transport of particles in the LPT model (presented in Figure 2). The LPT model follows the slope of conductivity and includes the diffusion term, which stochastically spreads the particles, as described by the diffusion dominance in the Péclet number. As the pulse of particles spreads, they start to distribute unevenly between different conductivity bins according to the conductivity slope, leading to different bin velocities and transforming the diffusive spread into a dispersion spread. However, even for high Péclet numbers, the outcome of the $F_{hp}$ criteria holds for either slope direction.

This transition effect on the transport is presented by the locations of the particles and when the first particle reaches$\;x_{j}=30$. The particles’ mean locations are reduced from each singular particle location to generate a location histogram ($Hist(x_{1..n}-\langle x_{1..n}\rangle )$) and extract the histogram variance (${\sigma_{F_{hp}}^{2}(x}_{1..n})$). This analysis allows us to visually compare particle dispersion since the center of each histogram is at zero (Figure 2).

This analysis shows that the dispersion of particles transitions from maximal (red curve in Figure 2a) to minimal (green, blue, and black curves in Figure 2a), respectively, following the head (Δh) transition from non-linear to linear (Figure 1c) and the transition of $F_{hp}$ from $F_{hp}>1$ to $F_{hp}\ll 1$. This transition from hyperbolic to parabolic flow corresponds, respectively, to an advection-dominated transport and a dispersion-dominated transport. As all particles enter the system together, the stochasticity of diffusion in the Langevin equation separates them and moves them between cells at different times, and as the Péclet number increases, this stochasticity decreases, leading to a reduction in dispersion. This reduction in dispersion is apparent in Figure 2b,c, where the head difference (∆h) increases from 2 to 10 and 100, respectively.

### 3.2. Breakthrough Curve and the Transition Between the Hyperbolic and Parabolic Flow Equations

**Figure 3:**
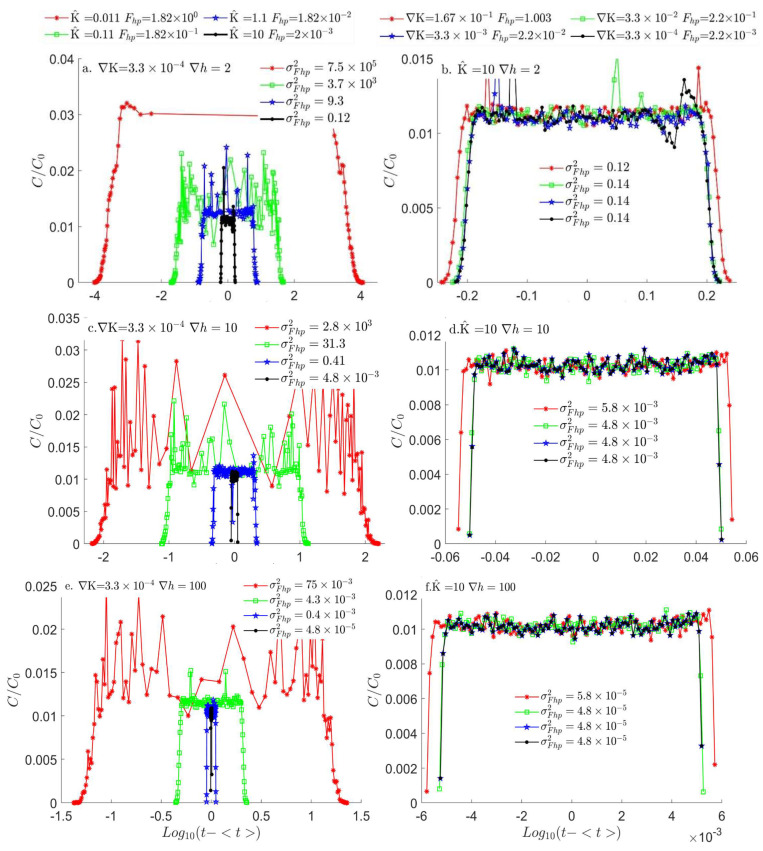
Normalized BTCs on a logarithmic scale as depicted in Equation (13) for constant slope value and varying mean hydraulic conductivity, with head differences of (**a**) ∆h=2, (**c**) ∆h=10, and (**e**) ∆h=100. For all cases, as the $F_{hp}$ decreases and the ∇h switches from hyperbolic to parabolic, the variance of the BTC distribution ($\sigma_{Fhp}^{2}$) decreases at a higher rate. Normalized BTCs for constant hydraulic conductivity value and varying slope, with head differences of (**b**) ∆h=2, (**d**) ∆h=10, and (**f**) ∆h=100. While the $F_{hp}$ is smaller than one and the ∇h is parabolic, the variance of the BTC distribution is constant, as the $F_{hp}$ rises above one and the ∇h changes to hyperbolic.

Using the same LPT simulations from Section 3.1. and extracting a histogram for the time each particle reaches the outlet boundary, the breakthrough curve (BTC) for the tracer particles is calculated (Figure 3). To compare the various BTCs for each parameter combination, the particles’ average time is reduced from each singular particle BTC time; however, as the range of the BTC mean value is a minimum of two orders of magnitude between each pair of BTCs, among the various ∇K and $\hat{K}$ combinations, the following logarithmic presentation of the temporal histogram is used to improve the visual distinction among the BTCs:

(14) $$BTC=\left\{\begin{array}{c} -\log_{10}\left(Hist\left(t_{1..n}-\langle t_{1..n}\rangle \right)\right),\;\;t<0\\ \log_{10}\left(Hist\left(t_{1..n}-\langle t_{1..n}\rangle \right)\right),\;\;t>0 \end{array}\right.,\;$$

while variance (${\sigma_{F_{hp}}^{2}(t}_{1..n})$) is extracted on the original BTCs.

Figure 3a presents the normalized concentration of BTC particles on a logarithmic scale as depicted in Equation (14) for a head difference of Δh = 2 as the slope is fixed on $\nabla K=3.3\times 10^{-4}$ and increases the mean hydraulic conductivity $\hat{K}$ from 0.011 to 10. For all head differences, the $F_{hp}$ decreases from 1.82 to $2\times 10^{-3}$. Figure 3a also shows a decrease in dispersion from maximal to minimal, manifested in the decrease in BTC variance (${\sigma_{F_{hp}}^{2}(t}_{1..n})$). This gradual decrease in variance is monotonical (~two orders of magnitude for each change) for $F_{hp}<1$. However, as $F_{hp}$ becomes bigger than one, there are more than three orders of magnitude changes in the variance due to the change in ∇h to non-linear (see Figure 3a). This change variance repeats for Δh=100 and 10, shown in Figure 3c,e, yet with a smaller magnitude due to the higher Pe.

When the mean hydraulic conductivity is fixed on $\hat{K}=10$ for identical head differences (Figure 3b,d,f for Δh = 2, 10, and 100, respectively), and the slope increases from $\nabla K=3.3\times 10^{-4}$ to $\nabla K=1.6\times 10^{-1}$, there is no change in variance for three orders of magnitude of $F_{hp}$ increase [$F_{hp}=0.2\times (10^{-2},\;10^{-1},\;\mathrm{and}\;10^{0})$] that are all < 1; however as $F_{hp}$ is slightly above 1, namely $F_{hp}=1.003$, the variance increases for all the head differences.

**Figure 4:**
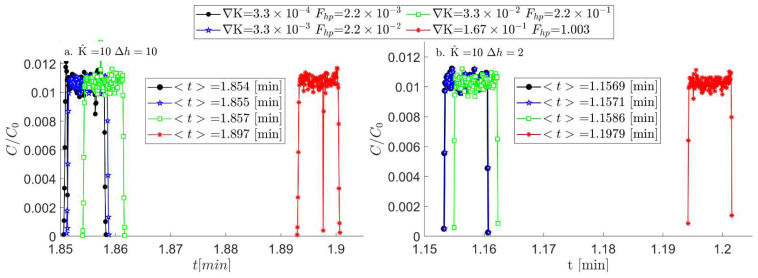
BTCs for (**a**) ∆h=10 and (**b**) ∆h=2 with constant mean hydraulic conductivity and different slope values. As the $F_{hp}$ decreases, turning the ∇h from hyperbolic to parabolic, the mean BTC time increases, yet there is an abrupt change in the BTC time for $F_{hp}>1$.

This sharp transition is also apparent in the non-normalized BTCs (Figure 4a,b), where there is a small monotonical increase in the BTC average time with the $F_{hp}$ increase until $F_{hp}>1,$ and the BTC mean value changes abruptly. This abrupt change in variance is due to the transition of ∇h to become non-linear. The sharp transition stems from the abrupt change in OLhLK, where ∆h changes linearly with the slope until $F_{hp}>1$ and therefore ∇h changes to non-linear and $L_{h}$ differs from $L_{K}$, which remains constant. This abrupt change in variance due to the change is apparent for all Pe values in our simulations (Figure 3), yet the variance increases as the Pe decreases and the relative magnitude of diffusion increases, thus exacerbating the dispersion.

The change in the variance of the BTCs indicates that there are similarities between the non-dimensional temporal nature of the Péclet number and the non-dimensional spatial nature of $F_{hp}$, in that they both characterize the magnitude of dispersion relative to the velocity. The Péclet number, which is a measure of the advection characteristic time scale to the diffusion characteristic time scale, affects the dispersion of particles over time following this time scale ratio. This similarity among the temporal nature of the Péclet number and the spatial nature of $F_{hp}$ is theoretically explored in the Supplementary Materials.

### 3.3. Tilled Soil and the Transition Between Hyperbolic and Parabolic Flow

**Figure 5:**
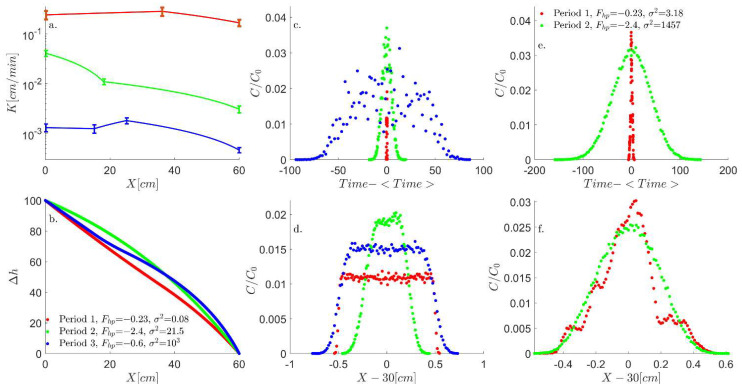
LPT simulation on interpolated soil conductivity measurements taken at three periods after tillage. Reproduced from Petersen et al. 2008 [33] (see Table 3). (**a**) Hydraulic conductivity spatial distribution exhibits a monotonic spatial change while (**b**) presents the head spatial distribution. Note that there is a transition between the parabolic head (nearly linear red curve) to hyperbolic (non-linear green curve) as the $F_{hp}$ changes from a fraction of 1 to being above 1, respectively, with the exception of the non-monotonic change in permeability (blue curve), which leads to a varying head distribution. This non-monotonic change leads to a large dispersion for Δh=100, in (**c**) the BTC and (**d**) spatial distribution, while the monotonic change, although non-linear, follows the $F_{hp}$ change for the BTC and the spatial distribution, marked by the low variance and sharp concentration increase and drop for the fractional $F_{hp}$, and the high variance and dispersive concentration increase and drop for the higher $F_{hp}.$ This dispersion and variance increase with the $F_{hp}$ change is more pronounced for Δh=10, in (**e**) the BTC variance increase, and (**f**) the spatial dispersion.

To examine the relevance of the $F_{hp}$ to field condition, the LPT simulation was applied on interpolated soil conductivity (see Equation (13) and Section 2.3) measurements taken at three periods after tillage, reproduced from [33]. As can be seen in Figure 5a, the interpolated hydraulic conductivity spatial distribution exhibits a monotonic yet non-linear spatial change for periods 1 and 2, yet period 3 is non-linear and non-monotonic. Nonetheless, the non-linear yet monotonic conductivity spatial change does lead to a transition between parabolic to hyperbolic head (Figure 5b, nearly linear green curve to non-linear red curve, respectively), as the $F_{hp}$ changes from $F_{hp}=-0.23$ to $F_{hp}=-2.4$, respectively. However, the non-monotonic conductivity spatial distribution for period 3 leads to a non-uniform head distribution (blue curve in Figure 5a and Figure 5b, respectively), which leads to an increase in the temporal and spatial dispersion for the BTC and spatial concentration (blue curve in Figure 5c and Figure 5d, respectively), showing that the $F_{hp}$ categorization is only applicable for monotonic change in conductivity.

Focusing on the monotonic yet non-linear change in conductivity for Δh=100 shows that both the BTC and the concentration spatial distribution follow the $F_{hp}$ categorization between parabolic to hyperbolic marked by the low variance ($\sigma^{2}=0.08$) and sharp concentration increase and drop for the fractional $F_{hp}$ (green curve in Figure 5c and Figure 5d, respectively) and the high variance ($\sigma^{2}=21.5$) and dispersive concentration increase and drop for the higher $F_{hp}$ (red curve in Figure 5c and Figure 5d, respectively). This dispersion and variance increase with the $F_{hp}$ change is more pronounced for Δh=10 for the BTC variance and concentration spatial distribution (Figure 5e and Figure 5f, respectively), in line with previous findings on the Péclet number effect.

### 3.4. Scaling of the Variance with the Head Difference

**Figure 6:**
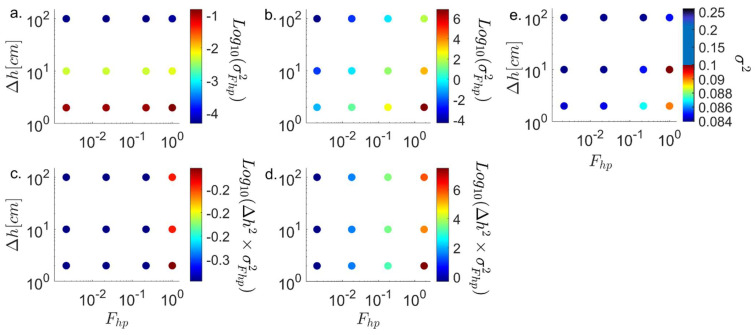
The temporal dispersion, accounted by the BTC $\sigma_{F_{hp}}^{2}$ for (**a**) constant $\hat{K}$ and varying ∇K and (**b**) constant ∇K and varying $\hat{K}$. Both show a clear transition in the variance with the $F_{hp}$ and ∆h, yet for the constant ∇K the head change is far more dominant. Multiplying the $\sigma_{F_{hp}}^{2}$ by the square head (${{∆h}^{2}\cdot \sigma }_{Fhp}^{2}$) for (**c**) constant $\hat{K}$ and varying ∇K and (**d**) constant ∇K and varying $\hat{K}$ normalizes the effect of ∆h completely for the first, or brings it to the same order for the latter. (**e**) The spatial dispersion, accounted by the $\sigma^{2}$ for constant ∇K and varying $\hat{K}$, shows a clear transition in the variance with the $F_{hp}$ and ∆h. Specific values are presented in Table 4.

Analyzing the variance of the particle spatial spread and variance of the particle temporal spread as a function of the $F_{hp}$ and ∆h (as depicted in Figure 2 and Figure 3, respectively) showed a noticeable change in the nature of these variances when the FE transitions from hyperbolic to parabolic, as the $F_{hp}$ changes across the threshold of one unit. Focusing on the variance of the particle’s spatial dispersion (Figure 6e), this change is more pronounced when ∆h is smaller due to the increased dominance of the stochastic diffusion component. Furthermore, this rate of change in the variance appears to follow changes in both $F_{hp}$ and ∆h, yet this change is more pronounced when the FE is in the hyperbolic regime and ∆h is smaller.

The variance of the BTC seems to change more dramatically as $F_{hp}$ is varied across the threshold of one unit for both fixed ∇K and $\hat{K}$. For the case of constant $\hat{K}$ and varying ∇K (see Table 4 and Figure 6a), the variance changes abruptly from a fixed value as $F_{hp}$ increases above one unit. In contrast, for the case of constant ∇K and varying $\hat{K}$ (see Figure 6b and Table 4), the variance changes monotonically with $F_{hp}$ for $F_{hp}$< 1 and non-monotonically for $F_{hp}$ > 1. In both cases, the magnitude of the change in variance is scaled by ∆h. When multiplying the variance by ${∆h}^{2}$ for the ∇K and $\hat{K}$ cases, the multiplied variance values (${{∆h}^{2}\cdot \sigma }^{2}$) become identical for all ∆h in the constant $\hat{K}$ case (Figure 6c and Table 4) and are of the same order as the constant ∇K case (Figure 6d and Table 4). This suggests that when $F_{hp}$ < 1, the dispersion, approximated by the variance and multiplied by ${∆h}^{2}$, provides a single value due to the linear trend of ∆h. This relationship between the increase in $\hat{K}$ or decrease in ∇K and the normalized variance comes from the order of magnitude analysis in Equation (7b), which shows that the ratio OLhLK remains the same for all parabolic values, as $L_{h}$ and $L_{K}$ are defined as the length of the field and $O\left(∆K\right)\neq O\left(\hat{K}\right)$. In contrast, for the hyperbolic case where $O\left(∆K\right)=O\left(\hat{K}\right)\approx 10$, the $L_{K}$ is a property of the field and remains the same, yet $L_{h}$ changes due to the non-linear change in ∆h and can be determined by the head difference multiplied by the differential change in the head over the field [$L_{h}=∆h/(\frac{dh}{dx})$]. For the hyperbolic linear case, this will be the length of the field, yet for the parabolic case, where $\frac{dh}{dx}$ is non-linear, fitting an exponential and derivating it on the field boundaries provides $L_{h}\approx 48.3$ for all ∆h, and the dominant ratio is OLhLK<1. The ratio of the hyperbolic to parabolic $L_{h}$ is the same ratio as the variance [$\frac{L_{h}(F_{hp}>1)}{L_{h}(F_{hp}<1)}\approx \frac{\sigma^{2}\left(F_{hp}>1\right)}{\sigma^{2}\left(F_{hp}<1\right)}=0.8$). This analysis of the $L_{h}$ defines it as the spatial length scale at which the slope of ∆h changes, or the curvature for ∆h. This should also be the case for $L_{K}=∆K/(\frac{dK}{dx})$], as the hydraulic conductivity may also change in a non-linear way. In any monotonical form, one can establish these length scales through this analysis.

## 4. Discussion

This study presents the effect of a monotonically varying heterogeneity on subsurface flow and transport by calculating a non-dimensional number that dictates the transition from hyperbolic to parabolic dominant forms of the saturated macroscopic flow equation (FE). This change can be traced to the slope and mean of the permeability as manifested in the hydraulic conductivity or to the length scale corresponding to the spatial change in the permeability versus the spatial change in ∆h. This derivation is accompanied by a 1D numerical solution demonstrating the abrupt change in ∆h from hyperbolic to parabolic FE and showing how this change leads to a change in the transport dispersion, affected by the Péclet number. There is also a similarity between the Péclet number, which relates the macroscopic temporal scale of advection and diffusion in the TE, and the $F_{hp}$ number, which discerns the macroscopic advection to the dispersion in the TE (manifested by the hyperbolic to parabolic FE) by the spatial scale for diverging (or sloping) conductivities (further analysis on the TE is in Section 3 in the Supplementary Materials).

This analysis presented here is useful when considering a monotonically changing hydraulic conductivity field, as it can explain how the mean hydraulic conductivity is less representative of the first passage time of a solute than the gradient of the hydraulic conductivity. Moreover, measuring the slope of hydraulic conductivity in the field using a Kozeny–Carmen approximation, or any other hydraulic conductivity calculation, can now provide the dominance of advection or dispersion with no need to solve the macroscopic transport equation. As shown in Section 3.3, this categorization may occur in real soils, and even the same soil measured at different periods, as was found for the interpolated tillage soil data [35]. This change from parabolic to hyperbolic is the outcome of the natural compaction mechanisms influencing the soil sampled at various periods, and as the simulation and $F_{hp}$ analysis show, this compaction has an effect on the dispersion and infiltration of solutes in saturated soil. This analysis on actual soil shows that it is the monotonicity of change that is important, and not necessarily the linearity; yet as this is but one analysis, further investigation is needed on the sensitivity.

While the focus of this study was on hydraulic conductivity monotonic change, which comes from geometrical considerations (i.e., permeability), the analysis was conducted on hydraulic conductivity, which is also a function of fluid viscosity and density. A recent study on miscible phase flow in porous media with fixed head difference and fixed geometry (constant permeability) shows the divergence of the flux and transport due to the evolving gradient of viscosity within the porous structure [54]. The quasistatic changes in flux as the viscosity gradient evolves during the phase mixing and invasion resemble the transition from a linear to non-linear change in head for this study, and the FE transition from hyperbolic to parabolic, as the hydraulic conductivity mean to slope ratio changes. Moreover, the dependency of the hydraulic conductivity change with the initial head difference has similar non-dimensional qualities to Equation (7a,b), which we intend to explore in a future study. These similarities show that this work is not only relevant to geometrical gradients, captured by the permeability, but also to conductivity gradients due to fluid mixing. Moreover, we intend to generalize our findings to cases where conductivity will monotonically change in miscible phase flow, as well as flow in peat formations.

## 5. Conclusions

The main conclusions presented in this work are as follows:

- As $F_{hp}$ switches from a number higher than one unit to a fracture, the FE switches from hyperbolic to parabolic forms, respectively, leading to a ∇h spatial transition from sharp (non-linear) to smooth (linear) and a change in the TE manifested in the variance change.
- The mechanism that allows the solute pulse to disperse due to the ∇h spatial transitions is the stochastic diffusion, which exacerbate or moderate the dispersion according to the $F_{hp}$ number, as shown by the Péclet number analysis.
- As the $F_{hp}$ represents the spatial contribution of the hydraulic conductivity slope, the Péclet represents the temporal influence of dispersion on transport, and both numbers are insensitive to the flow direction.
- This change in $F_{hp}$ can be moderated by the hydraulic conductivity representative length of the gradient, $L_{K},$ and the length represents the head change, $L_{h}$, leading to either a smooth change in transport dispersion as the mean hydraulic conductivity slope or an abrupt change in dispersion, as the slope changes.

These conclusions point to the role of monotonicity in the permeability structure on flow and transport at the Darcy scale. In this context, the hyperbolic FE can be evident in what is believed to be an almost impervious (e.g., heterogeneous) formation. Furthermore, these various aspects are shown for tillage and may have consequences for peat formation analysis and even miscible phase mixing at the Darcy scale. We acknowledge that to accurately implement our findings, a detailed knowledge is needed on the permeabilities as well as the head difference applied. However, these findings can still serve as a possible explanation to highly dispersive flow processes.

## Figures and Tables

**Table 1:** Hydraulic conductivity mean, slope, and head drop values used for the study of all of their combinations.

$\hat{K}$ [$\frac{cm}{min}$]	∇*K* [$\frac{cm}{min}$]	∆*h* [cm]
0.011	$3.3\times 10^{-4}$	2
0.11	$3.3\times 10^{-3}$	5
1.1	$3.3\times 10^{-2}$	10
10	0.16	100
100		

**Table 2:** Péclet number calculated for each hydraulic conductivity mean, slope, and head drop value used for this study.

Pe		$\hat{K}$[$\frac{cm}{min}$]			
		0.011	0.11	1.1	10
∆h [cm]	2	1.96	6.2	19	59
	10	4.38	13.8	43	132
	100	13.8	43.8	138	417

**Table 3:** Anisotropic soil conductivity (${\hat{K}}_{soil}$) measurements with respect to depth (D) taken at three periods after tillage. Reproduced from Petersen et al. 2008 [33]. Both the mean and standard deviation (STD) values were converted to cm/min.

Period (Months)		${\hat{K}}_{soil}$[$\frac{cm}{min}$], (*STD*)		
		1	8	32
D [cm]	0–5	0.24 (0.047)	-	0.0013 (2.32 × 10^−4^)
	10–15	0.28 (0.054)	0.04 (0.0053)	0.0012 (2.38 × 10^−4^)
	25	0.16 (0.025)	0.011 (0.0015)	0.0018 (2.39 × 10^−4^)
	40–60	-	0.0031 (0.0005)	4.65 × 10^−4^ (5.6 × 10^−5^)

**Table 4:** The specific dispersion values for Figure 5 for a. constant $\hat{K}$ and varying ∇K; b. constant ∇K and varying $\hat{K}$; normalized variance for c. constant $\hat{K}$ and varying ∇K; d. constant ∇K and varying $\hat{K}$; and e. the dispersion values for constant ∇K and varying $\hat{K}$.

		a. F_hp_ with fixed $\hat{K}=10$ $\left[\frac{cm}{min}\right]$			
		1.003	0.22	0.022	0.0022
∆h [cm]	2	0.15	0.12	0.12	0.12
	10	5.8 × 10^−3^	4.8 × 10^−3^	4.8 × 10^−3^	4.8 × 10^−3^
	100	5.8 × 10^−5^	4.8 × 10^−5^	4.8 × 10^−5^	4.8 × 10^−5^
		b. F_hp_ with fixed $\nabla \cdot K=3\times 10^{-4}\;[\frac{cm}{min}]$			
		1.82	0.182	0.0182	0.002
∆h [cm]	2	75.8 × 10^5^	370	9.3	0.12
	10	2.8 × 10^3^	31	0.41	4.8 × 10^−3^
	100	75	0.4	4.3 × 10^−3^	4.8 × 10^−5^
${{∆h}^{2}\cdot \sigma }_{Fhp}^{2}$		c. F_hp_ with fixed $\hat{K}=10$ $\left[\frac{cm}{min}\right]$			
		1.003	0.22	0.022	0.0022
∆h [cm]	2	0.6	0.48	0.48	0.48
	10	0.58	0.48	0.48	0.48
	100	0.58	0.48	0.48	0.48
${{∆h}^{2}\cdot \sigma }_{Fhp}^{2}$		d. F_hp_ with fixed $\nabla \cdot K=3\times 10^{-4}\;[\frac{cm}{min}]$			
		1.82	0.182	0.0182	0.002
∆h [cm]	2	3.03 × 10^7^	1480	37.2	0.48
	10	2.8 × 10^7^	3100	41	0.48
	100	7.5 × 10^7^	4000	43	0.48
		e. F_hp_ with fixed $\nabla \cdot K=3\times 10^{-4}\;[\frac{cm}{min}]$			
		1.82	0.182	0.0182	0.002
∆h [cm]	2	0.26	0.087	0.0847	0.0845
	10	0.092	0.085	0.084	0.0839
	100	0.085	0.084	0.08397	0.08395

## Data Availability

Code and data are available on a dedicated GitHub repository upon request to Yaniv Edery (yanivedery@technion.ac.il).

## Supplementary Materials

The following supporting information can be downloaded at https://www.mdpi.com/article/10.3390/e26110904/s1, Figure S1: Normalized particle concentration per location, as the first particle j reaches to *x_j_* = 30, for a slope of ∇*K* = −3.3 × 10^−4^, and heads a. 2, b. 10, and c. 100, with constant hydraulic conductivity slope which is negative, and different mean hydraulic conductivity, leading to negative *F_hp_*. As can be seen, regardless of the head value, the concentration variance is correlated with *F_hp_*, in the same way as for the positive *F_hp_*. d. Hydraulic conductivity spatial distribution for constant mean hydraulic conductivity, and different negative hydraulic conductivity slopes. e. Head spatial distribution for the same values of constant mean hydraulic conductivity and different negative hydraulic conductivity slopes as in d. Note the transition between parabolic head (linear slopes), and hyperbolic (exponential decay of the red curve). f. Similar transition between parabolic head (linear slopes), and hyperbolic (exponential decay of the red curve), is apparent for the case of constant negative hydraulic conductivity slope with different mean hydraulic conductivity, as the *F_hp_* transition below or above −1, respectively. Figure S2: Normalized BTC’s on a logarithmic scale as depicted in Equation (13), for constant negative slope value, and varying mean hydraulic conductivity, with head differences of a. ∆*h* = 2, c. ∆*h* = 10, and e. ∆*h* = 100. For all cases, as the negative F_hp_ decreases and the ∇*h* switches from hyperbolic to parabolic, the variance of the BTC’s distribution ($\sigma_{Fhp}^{2}$) decrease in higher rate. Normalized BTC’s for constant hydraulic conductivity value, and varying negative slope, with head differences of b. ∆*h* = 2, d. ∆*h* = 10, and f. ∆*h* = 100. While the F_hp_ is smaller than one and the ∇*h* is parabolic, the variance of the BTC’s distribution is constant, as the F_hp_ rises above one and the ∇*h* changes to hyperbolic, the variance increases abruptly. As can be seen, the change in slope direction did not change the overall behavior [55,56].

## References

[1] Bear J., Bachmat Y. (1990). Introduction to Modeling of Transport Phenomena in Porous Media.

[2] Bear J. (2013). Dynamics of Fluids in Porous Media.

[3] Cirpka O.A., Kitanidis P.K. (2000). Characterization of mixing and dilution in heterogeneous aquifers by means of local temporal moments. Water Resour. Res..

[4] Levy M., Berkowitz B. (2003). Measurement and analysis of non-Fickian dispersion in heterogeneous porous media. J. Contam. Hydrol..

[5] Kang P.K., Anna P., Nunes J.P., Bijeljic B., Blunt M.J., Juanes R. (2014). Pore-scale intermittent velocity structure underpinning anomalous transport through 3-D porous media. Geophys. Res. Lett..

[6] Edery Y., Dror I., Scher H., Berkowitz B. (2015). Anomalous reactive transport in porous media: Experiments and modeling. Phys. Rev. E.

[7] Gelhar L.W. (1986). Stochastic subsurface hydrology from theory to applications. Water Resour. Res..

[8] Cushman J.H., Ginn T.R. (1993). Nonlocal dispersion in media with continuously evolving scales of heterogeneity. Transp. Porous Media.

[9] Berkowitz B., Scher H. (1997). Anomalous transport in random fracture networks. Phys. Rev. Lett..

[10] Haggerty R., McKenna S.A., Meigs L.C. (2000). On the late-time behavior of tracer test breakthrough curves. Water Resour. Res..

[11] Morales-Casique E., Neuman S.P., Guadagnini A. (2006). Non-local and localized analyses of non-reactive solute transport in bounded randomly heterogeneous porous media: Theoretical framework. Adv. Water Resour..

[12] Le Borgne T., Dentz M., Carrera J. (2008). Lagrangian statistical model for transport in highly heterogeneous velocity fields. Phys. Rev. Lett..

[13] Bijeljic B., Mostaghimi P., Blunt M.J. (2011). Signature of non-Fickian solute transport in complex heterogeneous porous media. Phys. Rev. Lett..

[14] Sanchezvila X., Sánchez-Vila X., Carrera J. (2004). On the striking similarity between the moments of breakthrough curves for a heterogeneous medium and a homogeneous medium with a matrix diffusion term. J. Hydrol..

[15] Ciriello V., Guadagnini A., Di Federico V., Edery Y., Berkowitz B. (2013). Comparative analysis of formulations for conservative transport in porous media through sensitivity-based parameter calibration. Water Resour. Res..

[16] Roubinet D., De Dreuzy J.-R., Tartakovsky D.M. (2013). Particle-tracking simulations of anomalous transport in hierarchically fractured rocks. Comput. Geosci..

[17] Edery Y., Guadagnini A., Scher H., Berkowitz B. (2014). Origins of anomalous transport in heterogeneous media: Structural and dynamic controls. Water Resour. Res..

[18] Edery Y. (2021). The Effect of varying correlation lengths on Anomalous Transport. Transp. Porous Media.

[19] Dagan G., Neuman S.P. (2005). Subsurface Flow and Transport: A Stochastic Approach.

[20] Willmann M., Carrera J., Sánchez-Vila X. (2008). Transport upscaling in heterogeneous aquifers: What physical parameters control memory functions?. Water Resour. Res..

[21] Geiger S., Cortis A., Birkholzer J. (2010). Upscaling solute transport in naturally fractured porous media with the continuous time random walk method. Water Resour. Res..

[22] Jim Yeh T.-C. (1992). Stochastic modelling of groundwater flow and solute transport in aquifers. Hydrol. Processes.

[23] Neuman S.P. (2005). Trends, prospects and challenges in quantifying flow and transport through fractured rocks. Hydrogeol. J..

[24] Neuman S.P. (2020). Twenty lessons drawn from my subsurface hydrology career. Perspect. Earth Space Sci..

[25] Berkowitz B., Cortis A., Dentz M., Scher H. (2006). Modeling non-Fickian transport in geological formations as a continuous time random walk. Rev. Geophys..

[26] Hemond H.F., Goldman J.C. (1985). On non-Darcian water flow in peat. J. Ecol..

[27] Beckwith C.W., Baird A.J., Heathwaite A.L. (2003). Anisotropy and depth-related heterogeneity of hydraulic conductivity in a bog peat. I: Laboratory measurements. Hydrol. Process..

[28] Pharoah J.G., Karan K., Sun W. (2006). On effective transport coefficients in PEM fuel cell electrodes: Anisotropy of the porous transport layers. J. Power Sources.

[29] Iwanek M. (2008). A method for measuring saturated hydraulic conductivity in anisotropic soils. Soil Sci. Soc. Am. J..

[30] Wang J.-J., Qiu Z.-F. (2017). Anisotropic hydraulic conductivity and critical hydraulic gradient of a crushed sandstone–mudstone particle mixture. Mar. Georesources Geotechnol..

[31] Schoeneberger P., Amoozegar A. (1990). Directional saturated hydraulic conductivity and macropore morphology of a soil-saprolite sequence. Geoderma.

[32] Cai J., Taute T., Hamann E., Schneider M. (2015). An Integrated Laboratory Method to Measure and Verify Directional Hydraulic Conductivity in Fine-to-Medium Sandy Sediments. Groundwater.

[33] Petersen C., Trautner A., Hansen S. (2008). Spatio-temporal variation of anisotropy of saturated hydraulic conductivity in a tilled sandy loam soil. Soil Tillage Res..

[34] Lozano L.A., Soracco C.G., Cornelis W.M., Gabriels D., Sarli G.O., Villarreal R. (2013). Anisotropy of Pore Size Classes’ Con-nectivity Related to Soil Structure Under No Tillage. Soil Sci..

[35] Hong B., Li X., Wang L., Li L. (2019). Temporal variation in the permeability anisotropy behavior of the Malan loess in northern Shaanxi Province, China: An experimental study. Environ. Earth Sci..

[36] Pulido-Moncada M., Katuwal S., Munkholm L.J. (2022). Characterisation of soil pore structure anisotropy caused by the growth of bio-subsoilers. Geoderma.

[37] Holden J., Burt T.P. (2003). Hydraulic conductivity in upland blanket peat: Measurement and variability. Hydrol. Process..

[38] Lewis C., Albertson J., Xu X., Kiely G. (2012). Spatial variability of hydraulic conductivity and bulk density along a blanket peatland hillslope. Hydrol. Process..

[39] Holden J. (2006). Peatland hydrology. Developments Earth Surf. Process..

[40] Morris P.J., Baird A.J., Belyea L.R. (2015). Bridging the gap between models and measurements of peat hydraulic conductivity. Water Resour. Res..

[41] Liu H., Janssen M., Lennartz B. (2016). Changes in flow and transport patterns in fen peat following soil degradation. Eur. J. Soil Sci..

[42] Güven O., Molz F.J., Melville J.G. (1984). An analysis of dispersion in a stratified aquifer. Water Resour. Res..

[43] Fiori A., Dagan G. (2002). Transport of a passive scalar in a stratified porous medium. Transp. Porous Media.

[44] Dentz M., Carrera J. (2007). Mixing and spreading in stratified flow. Phys. Fluids.

[45] Tang D.W.S., Van Der Zee S. (2022). Macrodispersion and Recovery of Solutes and Heat in Heterogeneous Aquifers. Water Resour. Res..

[46] Matheron G., De Marsily G. (1980). Is transport in porous media always diffusive? A counterexample. Water Resour. Res..

[47] Sorek S., Braester C. An adaptive Eulerian-Lagrangian approach for the numerical simulation of unsaturated flow. Proceedings of the 6th International Conference FEWR.

[48] Sorek S., Braester C. (1988). Eulerian-Lagrangian formulation of the equations for groundwater denitrification using bacterial activity. Adv. Water Resour..

[49] Edery Y., Stolar M., Porta G., Guadagnini A. (2021). Feedback mechanisms between precipitation and dissolution reactions across randomly heterogeneous conductivity fields. Hydrol. Earth Syst. Sci. Discuss..

[50] Shavelzon E., Edery Y. (2023). Shannon Entropy of Transport Self-Organization due to Dissolution/Precipitation Reaction at Varying Peclet Number in an Initially Homogeneous Porous Media. Hydrol. Earth Syst. Sci. Discuss..

[51] Dagan A., Edery Y. (2024). Bifurcating paths: The relation between preferential pathways, channel splitting, under sampled regions, and tortuosity on the Darcy scale. Adv. Water Resour..

[52] Guadagnini A., Neuman S.P. (1999). Nonlocal and localized analyses of conditional mean steady state flow in bounded, randomly nonuniform domains: 1. Theory and computational approach. Water Resour. Res..

[53] Domenico P., Schwartz F., D N. (1990). Physical and Chemical Hydrogeology.

[54] Eliyahu-Yakir Y., Abezga L., Edery Y. (2023). From mixing to displacement of miscible phases in porous media: The role of heterogeneity and inlet pressure. Phys. Rev. Fluids.

[55] Sorek S., Borisov V. (2012). Modified Eulerian–Lagrangian Formulation for Hydrodynamic Modeling. J. Comput. Phys..

[56] Huang K., Zhang R., Van Genuchten M.T. (1994). An Eulerian-Lagrangian Approach with an Adaptively Corrected Method of Characteristics to Simulate Variably Saturated Water Flow. Water Resour. Res..

