# Supplementary material for: On the Scaling of Transport Phenomena at a Monotonously Changing Hydraulic Conductivity Field
Source: Entropy (Basel). 2024 Oct 24;26(11):904. doi: 10.3390/e26110904 (PMC11593263; doi:10.3390/e26110904)
Supplement: Supplementary file 1 [file entropy-26-00904-s001.zip › entropy-3176320-supplementary.pdf]

SUPPORTING INFORMATION

In the supporting information we will set the generalized formulation framework (section 1.), which while it is standard it is important to present for completeness. We will then follow by a macroscopic consideration of the transport equation to interpret the F_{hp} resemblance to the Peclet number (section 2). We will also present the insensitivity of the F_{hp} to the change in slope direction in section 3.

1. Formulation framework

In this section will show that the F_{hp} derived from a deterministic theoretical framework, on the premise of continuum mechanics, scales the stochastic transport properties. Here, and extended in the supporting information, we elaborate on fundamental motion aspects in terms of Eulerian and Lagrangian (for particle tracking with which transport phenomena is assessed) formulation that comply with formation spatial heterogeneity. Let a phase γ ($[\ \ ]_\gamma = \sum_{\alpha=1}^{N}[\ \ ]_\gamma^\alpha$) be the sum of α components; phase extensive quantity E [e.g., mass ($E \equiv m \equiv m_\gamma = \sum_{\alpha=1}^{N} m_\gamma^\alpha$), volume, momentum, energy] being bounded within, U, phase volume; intensive quantity e ($e = dE/dU$). In what follows, vector/tensor variables are denoted in bold fonts, otherwise, these express scalar magnitudes. Position $\boldsymbol{X}^E$ is of a particle carrying E along a pathline with a velocity that is spatially referenced to the location $\boldsymbol{\xi}^E$, at an initial time relative to that fixed frame of reference ($\boldsymbol{\vartheta}^E = \left[\partial \boldsymbol{X}^E/\partial t\right]_{\boldsymbol{\xi}^E}$). From these parameters, the flux $\boldsymbol{J}^E$ of E can be written as $\boldsymbol{J}^E = e\boldsymbol{\vartheta}^E$. As $\boldsymbol{\vartheta}^E$ and $\boldsymbol{J}^E$ are detecTable only for E being volume, we rewrite $\boldsymbol{J}^E$ using $e\boldsymbol{\vartheta}^m$ the mass advective flux and $\boldsymbol{J}^{Em} \equiv e(\boldsymbol{\vartheta}^E - \boldsymbol{\vartheta}^m)$ the mass diffusive flux, as:

$$\boldsymbol{J}^E = e\boldsymbol{\vartheta}^E + e\boldsymbol{\vartheta}^m - e\boldsymbol{\vartheta}^m = e\boldsymbol{\vartheta}^m + e(\boldsymbol{\vartheta}^E - \boldsymbol{\vartheta}^m)\,, \qquad \text{(S1)}$$

in which $\boldsymbol{\vartheta}^m$ denotes the γ phase mass-weighted velocity obtained by,

$$m\boldsymbol{\vartheta}^m = \sum_{\alpha=1}^{N} m^\alpha \boldsymbol{\vartheta}^{m^\alpha}\,. \qquad \text{(S2)}$$

Following Eq. (S2) for any E weighted velocity $\boldsymbol{v}$ in view of Eq. (S1), we write,

$\boldsymbol{J}^E = e\boldsymbol{v} + \boldsymbol{J}$ in which $\boldsymbol{J} \equiv \boldsymbol{e}(\boldsymbol{\vartheta}^E - \boldsymbol{v})$ denotes the diffusive flux replaced by a constitutive relation in the form of an empirical potential flux $\boldsymbol{J} = -\boldsymbol{\mathfrak{D}} \cdot \boldsymbol{\nabla} e$ where $\boldsymbol{\mathfrak{D}}$ (a scalar or tensor) represents the

specific observation, e.g., Fick's law for component's mass diffusion flux, momentum diffusion flux (i.e., stress tensor term) for viscous fluid addressing Newtonian shear, or for elastic solid by Hook's stress, or for Fourier's law for energy diffusion flux.

Using the microscopic conservation (interchangeable with balance) equation for E per a unit of the control volume, with a mass-specific source Γ^E and mass density ρ, we can write the following in Eulerian form,

$$ {}^{\partial \mathrm{e}}\!/_{\partial t} + \boldsymbol{\nabla} \cdot (e\boldsymbol{v} + \boldsymbol{J}) - \rho \Gamma^E = 0, \qquad \text{(S3)} $$

Our focus is to elaborate on the effect of geometric monotonic heterogeneity on flow and transport, we thus refer to the hydraulic conductivity (K $[\frac{L}{T}]$) which is indicative to the degree of resistance to flow due to fluid properties and the formation geometrical characteristics marked by the permeability k [L^2]. To do this we need to refer to Lagrangian balance equation in the supporting information. Hence, Eq. (S3) in Lagrangian form for a particle carrying e along a path $\boldsymbol{x}$ reads,

$$ {}^{D_v}\!/_{Dt}(e) + e\boldsymbol{\nabla} \cdot \boldsymbol{v} + \boldsymbol{\nabla} \cdot \boldsymbol{J} - \rho \Gamma^E = 0, $$

$$ {}^{D_v}\!/_{Dt}(\boldsymbol{x}) = \boldsymbol{v}\,, \qquad {}^{D_v}\!/_{Dt} \equiv {}^{\partial}\!/_{\partial t} + \boldsymbol{v} \cdot \boldsymbol{\nabla}\,. \qquad \text{(S4)} $$

In order to obtain the macroscopic framework, we apply spatiotemporal averaging over the REV volume V that accounts for at least two phases, namely β and γ (e.g., β ≡ S designates solid and γ ≡ f that of fluid) with their corresponding V_β and V_γ alongside with their volume ratios θ_β and θ_γ (for a medium saturated with fluid, these volume ratios are associated with the porosity ϕ), spatial derivative and time rate that addresses the spatial averaged phase within the REV volume and interaction between β and γ across their common microscopic $S_{\beta\gamma}$ interface surface. Further,

we assume that the medium can be addressed with typical (effective) macroscopic quantities, e.g., the hydraulic conductivity $\mathbf{K}$ tensor and the hydrodynamic dispersive $\boldsymbol{D}_h$ tensor.

In what follows, we address field cases for which practical change in matrix porosity is assumed negligible. Focusing on geometrical monotonic heterogeneity, the supporting information addresses the non-fixed porosity. By Terzaghi's empirical law for the stress of a saturated bulk accounting for $\boldsymbol{\sigma}'_s$, the effective stress (i.e., associated with bulk deformation), and for non-constant porosity, given that there is no change in $\boldsymbol{\sigma}_b$ bulk stress (namely that $d\boldsymbol{\sigma}_b = d\boldsymbol{\sigma}'_s - dp\boldsymbol{I} = \mathbf{0}$, where p and $\boldsymbol{I}$ denote the pressure and the identity tensor, respectively) we can obtain that $\phi = \phi(p)$. This is formulated in the following section, where one reads how it affects a transport equation (TE).

2. Macroscopic transport equation

Let us consider a monotonically changing hydraulic conductivity field ($\nabla K \neq 0$) of an incompressible solid medium ($d\rho_s/dt = 0$) that is saturated with practically no change in its bulk stress [$\phi = \phi(p)$, as mentioned in the previous section], and a solute component (α) with low solubility that is subject to a linear equilibrium adsorption isotherm and is carried by Darcy's flow. No fluid or solute mass source terms, all are involved, and isotropic conditions prevail.

The role of F_{hp}, as part of the solute's spatiotemporal pattern distribution, is discussed in what follows.

The TE for α, following the Eulerian form (Eq. S3), is

$$\partial/_{\partial t}\{[\phi + (1-\phi)\rho_S\kappa_d]C\} = -\boldsymbol{\nabla}\cdot[\phi(C\boldsymbol{\vartheta} - \boldsymbol{D_h}\cdot\boldsymbol{\nabla}C)]. \qquad \text{(S5)}$$

The right-hand side of Eq. S5 addresses advective and dispersive mass fluxes, where the domination relationship between the two is addressed by P_{e_α} (Péclet number), which for α quantities is defined as,

$$P_{e_\alpha} = \frac{L_{C_\alpha} \vartheta_\alpha}{D_{h_\alpha}} . \tag{S6}$$

Here, L_{C_α} denotes the typical distance associated with the concentration gradient ∇C, and ϑ_α denotes the component's typical displacement velocity, which can be focused upon when writing the equation in its Lagrangian form (Eq. S4).

Following Darcy's Law (Eq. 1 in Edery and Sorek), the right-hand side of Eq. S5 is rewritten:

$$-\boldsymbol{\nabla} \cdot \left(C\, \phi \boldsymbol{\vartheta}_f - \boldsymbol{D}_h \cdot \phi \nabla \mathrm{C} \right) = [\boldsymbol{\nabla} \cdot \mathbf{K} \cdot \nabla h + (\mathbf{K} \cdot \boldsymbol{\nabla}) \cdot \boldsymbol{\nabla} h] C - \left(\phi \boldsymbol{\vartheta}_f - \phi \boldsymbol{\nabla} \cdot \boldsymbol{D}_h - \boldsymbol{\nabla} \mathbf{p} \cdot \boldsymbol{D}_h \right) \cdot \boldsymbol{\nabla} \mathrm{C} + \phi (\boldsymbol{D}_h \cdot \boldsymbol{\nabla}) \cdot \boldsymbol{\nabla} \mathrm{C}. \tag{S7}$$

In view of the Lagrangian form Eq. S4 and Eq. S5, we note that the apparent component velocity, $\boldsymbol{\vartheta}_\alpha$, is obtained by

$$\boldsymbol{\vartheta}_\alpha \equiv \phi \boldsymbol{\vartheta}_f - \phi \boldsymbol{\nabla} \cdot \boldsymbol{D}_h - \boldsymbol{\nabla} \mathbf{p} \cdot \boldsymbol{D}_h, \tag{S8}$$

from which we deduce the effect of heterogeneity associated with $\boldsymbol{D}_h$ in attenuation of the fluid velocity $\boldsymbol{\vartheta}_f$. Moreover, in view of Eq. 5.1 in Edery and Sorek, when subject to Eq. 7 in Edery and Sorek and Eq. S6 together with Eq. S8, the component of mass advective for a single particle becomes dominant, generating a sharp transition between the spatiotemporal velocities of all particles. For an ensemble of point source particles, however, stochastic diffusion will lead to spatial separation as they flow, and while every single particle will experience the same sharp velocity transition, the resulting stochastic dispersion for the particle ensemble will increase, as described by Eqs. 5.1 and 5.2 in Edery and Sorek. The ensemble has typical (i.e. magnitude only) quantities associated with the medium heterogeneity that become

$$\nabla \mathrm{K} \, \nabla h \gg K\nabla^2 h \,, \tag{S9}$$

and/or for,

$$L_{C_\alpha}\left(\phi\vartheta_f - \phi\nabla D_h - \nabla \mathrm{p}\, D_h\right) \gg D_{h_\alpha}. \tag{S10}$$

Furthermore, we note that by following the Lagrangian form (Eq. S4), we can rewrite the flow equation (Eq. 2 in Edery and Sorek) in a Lagrangian form to read,

$$S_S \frac{D_{\vartheta_h}}{Dt}(h) = K\nabla^2 h, \tag{S11}$$

according to which a particle carrying h will move with a velocity $\boldsymbol{\vartheta}_h$, given by

$$\boldsymbol{\vartheta}_h \equiv - \frac{\boldsymbol{\nabla}\cdot\mathbf{K}}{S_S} \,. \tag{S12}$$

Thus, according to Eq. S12, not only do we analyze the role of $\boldsymbol{\nabla}\cdot\mathbf{K}$**,** but also in view of F_{hp} and Eqs. S6 and S12, we reveal the equivalent similarity:

$$\frac{L_h S_S \vartheta_h}{K} \sim \frac{L_{C_\alpha}\vartheta_\alpha}{D_{h_\alpha}}\,, \tag{S13}$$

which enables to estimate the value of the $\frac{advection}{dispersion}$ ratio for the flow equation, which refers to the proportion between a single particle carrying a mass in a Lagrangian form (left-hand side of Eq. S13) and the transport equation (right-hand side of Eq. S13).

Sorek and Borisov, [1S], present a development similar to Eqs. S11 and S12 in which Hubert's potential is used in conjunction with the unsaturated flow equation, that can be solved using a particle tracking method. Huang et al., [2S], introduce the notion, following Sorek and Braester, [49], that a flow equation like Eq. 2 in Edery and Sorek has a parabolic-hyperbolic nature and can, therefore, be rewritten in Lagrangian form like Eq. S11, to be solved numerically using an apparent particle tracking velocity, like Eq. S12, unlike the fluid Darcian velocity.

Let us assess the role of the F_{hp} number, or the left-hand side of Eq. S13, in the P_{e_α} number in Eq. S6. In doing so, and without affecting the generality of the development, let us assume that the

fluid velocity, $\boldsymbol{\vartheta}_f$, is the dominant contributor to the $\boldsymbol{\vartheta}_\alpha$ velocity. Hence, Eq. S10 is now approximated to

$$\boldsymbol{\vartheta}_\alpha \cong \phi\boldsymbol{\vartheta}_f \ , \tag{S14}$$

and, in view of Eq. S14, the Péclet number from Eq. S6 is rewritten accordingly as

$$P_{e_\alpha} \cong \frac{L_{C_\alpha}(\phi\vartheta_f)}{D_{h_\alpha}} \ . \tag{S15}$$

Note that the left-hand side of Darcy's Law (Eq. 1 in Edery and Sorek) can be rewritten as,

$$\begin{aligned} -\boldsymbol{K}\cdot\boldsymbol{\nabla}h &= -\boldsymbol{\nabla}\cdot(\boldsymbol{K}\boldsymbol{h}) + (\boldsymbol{\nabla}\cdot\boldsymbol{K})h = -[(\boldsymbol{\nabla}\cdot\boldsymbol{K})h + \boldsymbol{K}\cdot\boldsymbol{\nabla}h] + (\boldsymbol{\nabla}\cdot\boldsymbol{K})h \\ &= -[\mathrm{h}\nabla K(\boldsymbol{\nabla}\cdot\boldsymbol{K}\,\boldsymbol{h})^* + K\nabla h(\boldsymbol{K}\cdot\boldsymbol{\nabla}h)^*] + (\boldsymbol{\nabla}\cdot\boldsymbol{K})h \\ &= -K\nabla h\left[\frac{L_h\nabla K}{K}(\boldsymbol{\nabla}\cdot\boldsymbol{K}\,\boldsymbol{h})^* + (\boldsymbol{K}\cdot\boldsymbol{\nabla}h)^*\right] + (\boldsymbol{\nabla}\cdot\boldsymbol{K})h \ , \end{aligned} \tag{S16}$$

where $(\ \ )^*$ denotes a value of a unit order and $L_h \cong h/\nabla h$ (complies with $L_h = \Delta h/(\frac{dh}{dx})$ of Section 3.3 in Edery and Sorek).

Hence, under the condition expressed by Darcy's Law and in view of Eq. S15, we obtain

$$(\boldsymbol{\nabla}\cdot\boldsymbol{K})h \gg \boldsymbol{K}\cdot\boldsymbol{\nabla}h \ . \tag{S17}$$

By virtue of Eqs. S12 and S17, we can assume that

$$\|\boldsymbol{\vartheta}_h\| \gg \|\phi\boldsymbol{\vartheta}_f\| \ , \tag{S18}$$

which dictates the influence of the K slope compared with its mean when introduced into the Péclet number in Eq. S15 through Eq. S18.

3. Analysis for negative slope

The same numerical analysis presented in Edery and Sorek is presented here for a negative slope, which provides a negative F_{hp}, with no significant change in our findings. This shows that the

transition from hyperbolic to parabolic depends only on the absolute value of F_{hp}, as stated in Eq. 6 in Edery and Sorek. See Figures S1 and S2 for details.

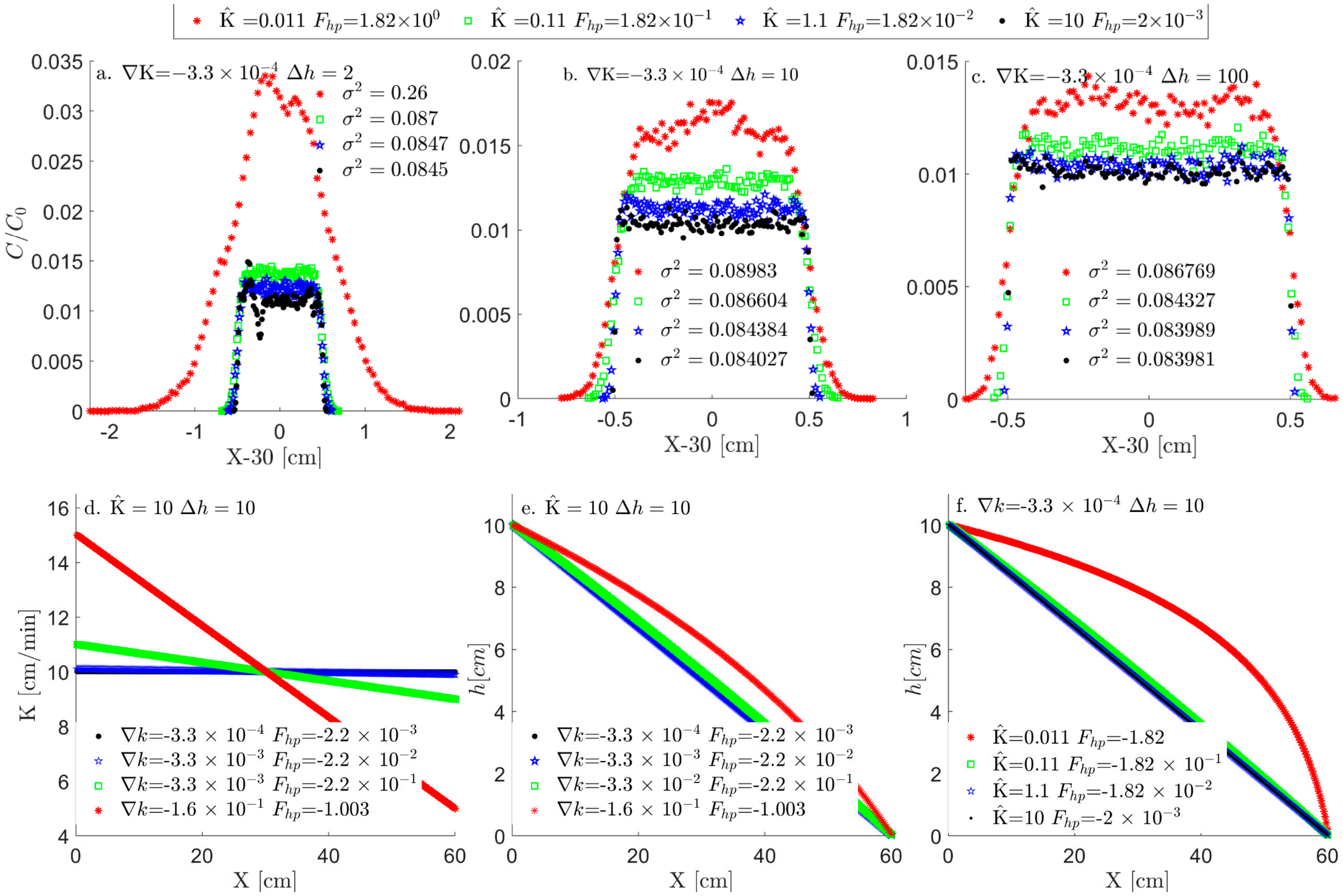

Figure S1. Normalized particle concentration per location, as the first particle j reaches to $x_j = 30$, *for a slope of* $\nabla K = -3.3 \times 10^{-4}$, *and heads a.2, b.10, and c.100, with constant hydraulic conductivity slope which is negative, and different mean hydraulic conductivity, leading to negative* F_{hp}. *As can be seen, regardless of the head value, the concentration variance is correlated with* F_{hp}, *in the same way as for the positive* F_{hp}.
d. Hydraulic conductivity spatial distribution for constant mean hydraulic conductivity, and different negative hydraulic conductivity slopes. e. Head spatial distribution for the same values of constant mean hydraulic conductivity and different negative hydraulic conductivity slopes as in d. Note the transition between parabolic head (linear slopes), and hyperbolic (exponential decay of the red curve). f. Similar transition between parabolic head (linear slopes), and hyperbolic (exponential decay of the red curve), is apparent for the case of constant negative hydraulic conductivity slope with different mean hydraulic conductivity, as the F_{hp} *transition below or above -1, respectively.*

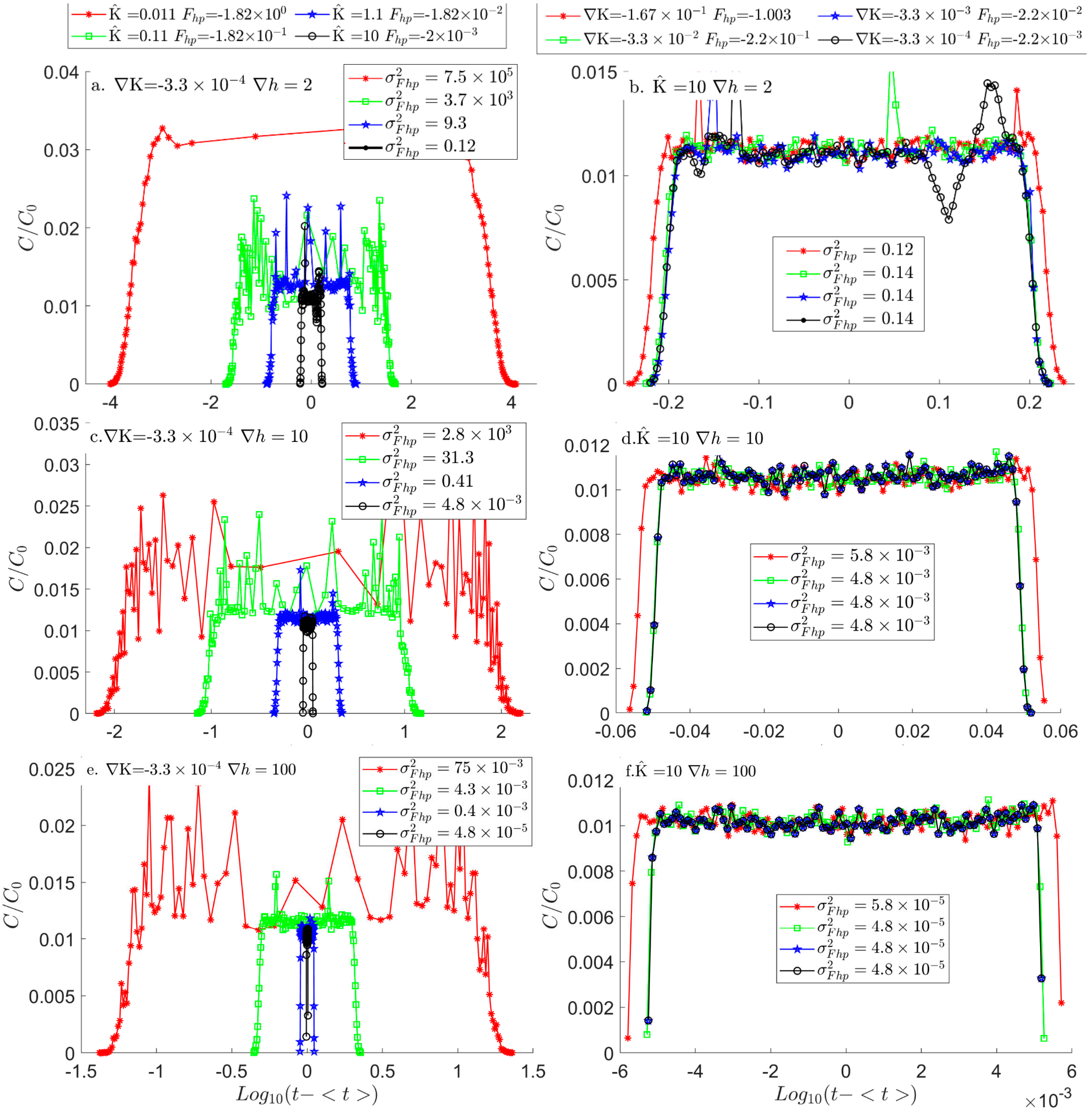

Figure S2. Normalized BTC's on a logarithmic scale as depicted in Eq. (13), for constant negative slope value, and varying mean hydraulic conductivity, with head differences of a. $\Delta h = 2$*, c.* $\Delta h = 10$*, and e.* $\Delta h = 100$*. For all cases, as the negative* F_{hp} *decreases and the* ∇h *switches from hyperbolic to parabolic, the variance of the BTC's distribution (*σ^2_{Fhp}*) decrease in higher rate. Normalized BTC's for constant hydraulic conductivity value, and varying negative slope, with head differences of b.* $\Delta h = 2$*, d.* $\Delta h = 10$*, and f.* $\Delta h = 100$*. While the* F_{hp} *is smaller than one and the* ∇h *is parabolic, the variance of the BTC's distribution is constant, as the* F_{hp} *rises above one and the* ∇h *changes to hyperbolic, the variance increases abruptly. As can be seen, the change in slope direction did not change the overall behavior.*

References

1S. S. Sorek and V. Borisov, *Modified Eulerian–Lagrangian Formulation for Hydrodynamic Modeling*, J. Comput. Phys., 2012, 231, 3083.

2S. K. Huang, R. Zhang, and M. T. van Genuchten, *An Eulerian-Lagrangian Approach with an Adaptively Corrected Method of Characteristics to Simulate Variably Saturated Water Flow*, Water Resour. Res., 1994, 30, 499.
